# A Cost-Effectiveness Tool to Guide the Prioritization of Interventions for Rheumatic Fever and Rheumatic Heart Disease Control in African Nations

**DOI:** 10.1371/journal.pntd.0004860

**Published:** 2016-08-11

**Authors:** David Watkins, Solomon J. Lubinga, Bongani Mayosi, Joseph B. Babigumira

**Affiliations:** 1 Division of General Internal Medicine, Department of Medicine, University of Washington, Seattle, Washington, United States of America; 2 Department of Medicine, Groote Schuur Hospital and University of Cape Town, Cape Town, South Africa; 3 Department of Global Health, University of Washington, Seattle, Washington, United States of America; 4 Department of Pharmacy, University of Washington, Seattle, Washington, United States of America; George Washington University, UNITED STATES

## Abstract

**Background:**

Rheumatic heart disease (RHD) prevalence and mortality rates remain especially high in many parts of Africa. While effective prevention and treatment exist, coverage rates of the various interventions are low. Little is known about the comparative cost-effectiveness of different RHD interventions in limited resource settings. We developed an economic evaluation tool to assist ministries of health in allocating resources and planning RHD control programs.

**Methodology/Principal Findings:**

We constructed a Markov model of the natural history of acute rheumatic fever (ARF) and RHD, taking transition probabilities and intervention effectiveness data from previously published studies and expert opinion. Our model estimates the incremental cost-effectiveness of scaling up coverage of primary prevention (PP), secondary prevention (SP) and heart valve surgery (VS) interventions for RHD. We take a healthcare system perspective on costs and measure outcomes as disability-adjusted life-years (DALYs), discounting both at 3%. Univariate and probabilistic sensitivity analyses are also built into the modeling tool. We illustrate the use of this model in a hypothetical low-income African country, drawing on available disease burden and cost data. We found that, in our hypothetical country, PP would be cost saving and SP would be very cost-effective. International referral for VS (e.g., to a country like India that has existing surgical capacity) would be cost-effective, but building in-country VS services would not be cost-effective at typical low-income country thresholds.

**Conclusions/Significance:**

Our cost-effectiveness analysis tool is designed to inform priorities for ARF/RHD control programs in Africa at the national or subnational level. In contrast to previous literature, our preliminary findings suggest PP could be the most efficient and cheapest approach in poor countries. We provide our model for public use in the form of a Supplementary File. Our research has immediate policy relevance and calls for renewed efforts to scale up RHD prevention.

## Author Summary

Rheumatic heart disease is a major cause of cardiovascular morbidity and mortality in Africa. Although there are effective medications and surgical procedures for rheumatic heart disease, they are under-used. What is more, these interventions can be expensive—even if they are feasible and effective. Unfortunately, there are currently very few economic studies on rheumatic heart disease, leaving ministries of health with little guidance on how to choose among various interventions and allocate resources to control programs. Our study describes the methods and data we used to develop a cost-effectiveness analysis tool that was intended specifically for decision-making in African countries. In our study, we also illustrate, in a hypothetical low-income African country, how the tool could be used. In our illustrative example, a prevention-oriented approach would save money in the long term, although other interventions could be cost-effective and feasible if enough financial resources were present. These findings contrast with previous studies and make a strong case that rheumatic heart disease prevention could be a high-priority intervention in Africa. We are making our tool publicly available and anticipate that ministries of health will use it as they develop or expand their rheumatic heart disease control programs.

## Introduction

Decision-makers in African countries face difficult tradeoffs when choosing among interventions that address acute rheumatic fever (ARF) and its sequel rheumatic heart disease (RHD). There is evidence that ARF and RHD can be eradicated in both high-income and limited resource settings.[1–3] Yet these conditions remain neglected by the global health community.[4] Further, the prevalence of RHD appears to be increasing, and mortality rates in Africa are among the highest in the world.[4,5]

ARF can usually be prevented by treating cases of streptococcal pharyngitis promptly with injectable benzathine penicillin G (“primary prevention”). Among individuals with a history of ARF, regular prophylactic use of penicillin can reduce the risk of recurrent ARF recurrence and progression to RHD (“secondary prevention”).[6] However, for many children and adults living with RHD in African countries, opportunities for prevention have been lost. Irreversible heart valve damage from RHD carries a high mortality rate that can only be mitigated by open heart surgery and valve replacement.[6,7]

Tradeoffs between prevention and surgical treatment of RHD are especially stark in settings where there is currently no capacity to perform specialized cardiac surgery. At present, only a handful of African nations have independent, high-volume surgical programs. While some nations have semi-independent or low-volume surgical centers, most nations have no surgical capacity.[8] At the same time, coverage rates of (relatively) more affordable primary and secondary prevention measures are also unacceptably low, even in academic referral hospitals and in middle-income countries.[9] Hence the central question for RHD health policy in Africa is how to set priorities across prevention and treatment interventions in order to maximize the health of individuals at risk of, or affected by, ARF and RHD.

At the same time, the global universal health coverage (UHC) movement has gained traction in light of the new Sustainable Development Goals (SDGs), which explicitly call for countries to achieve UHC over the SDG period.[10] These goals add urgency to the need to set country-specific priorities around ARF and RHD by identifying which health care services could feasibly be included in a UHC “benefits package” at an acceptable cost.[11] A WHO consultation recently identified the most fair and equitable pathway to UHC as one that starts by providing full population coverage at zero patient cost for a focused set of services that preferentially improve health for the worst off.[12] Since RHD is a highly preventable disease of poverty, ARF/RHD interventions need to be considered as part of UHC benefits packages for African nations—especially for UHC schemes that strive to be “pro-poor,” i.e., that focus their initial efforts on improving health and economic outcomes among the poor. One goal of economic evaluation, then, is to identify which ARF/RHD services are most cost-effective and affordable and should thus receive first priority for a benefits package.

There is scarce information on the cost-effectiveness of ARF and RHD interventions. Some have studied the most cost-effective method(s) of delivering primary prevention,[13] while others have studied whether echocardiography is a cost-effective tool for strengthening secondary prevention through “active” case-finding.[14] Only one analysis, which was undertaken for the original Disease Control Priorities in Developing Countries project (“DCP1”) in 1993, explicitly studied the policy question of how to choose between prevention and surgical treatment.[15] Yet the literature on the epidemiology of RHD, cost of care, and effectiveness of treatment have evolved substantially since that time, necessitating an update of those findings.

This study builds on the prior DCP1 analysis, incorporating up-to-date data and following contemporary modeling practices. The output of this work is a cost-effectiveness tool that decision-makers can use to allocate scarce resources for RHD efficiently at the local and national level as they consider the move towards UHC. In addition to presenting the methods and data sources for our model, we illustrate its use in a hypothetical low-income country setting.

## Methods

We adhere to the Consolidated Health Economic Evaluation Reporting Standards (CHEERS) statement in reporting the methods and results of our analysis.[16]

### Evaluation approach

The objective of our tool is to evaluate the incremental cost-effectiveness of achieving target coverage rates of one or more evidence-based interventions for ARF and RHD. We define coverage as the proportion of at-risk or affected individuals who are currently receiving (“ante”) or are intended to receive (“post”) the interventions. Our focus on incremental changes in coverage rates situates our analysis within the broader question of setting priorities for UHC in Africa.[17]

We model the health gains and costs associated with a given increase in coverage for a reference individual, and in the base case, we present an incremental cost-effectiveness ratios (ICER) for increased coverage. Our modeling tool looks at three general intervention scenarios:

1. Scaling up primary prevention (PP) services. This involves improving treatment of pharyngitis in primary care settings.
2. Scaling up secondary prevention (SP) services. This involves creating and maintaining a registry of individuals with a history of ARF or RHD who then receive prophylactic penicillin on a regular basis.
3. Increasing coverage of valve surgery (VS). This might involve building local surgical capacity de novo or increasing use of existing surgical services.

The reference case for PP is the general population, whereas the reference case for SP is the individual with a history of ARF who remains at risk of ARF recurrence and progression to RHD. The reference case for VS is the individual with pre-existing RHD. The ICER in each scenario then reflects the value for money for achieving full coverage of the intervention described in that scenario.

### Disease model and health-related inputs

#### Model for interventions and outcomes

**Fig 1:**
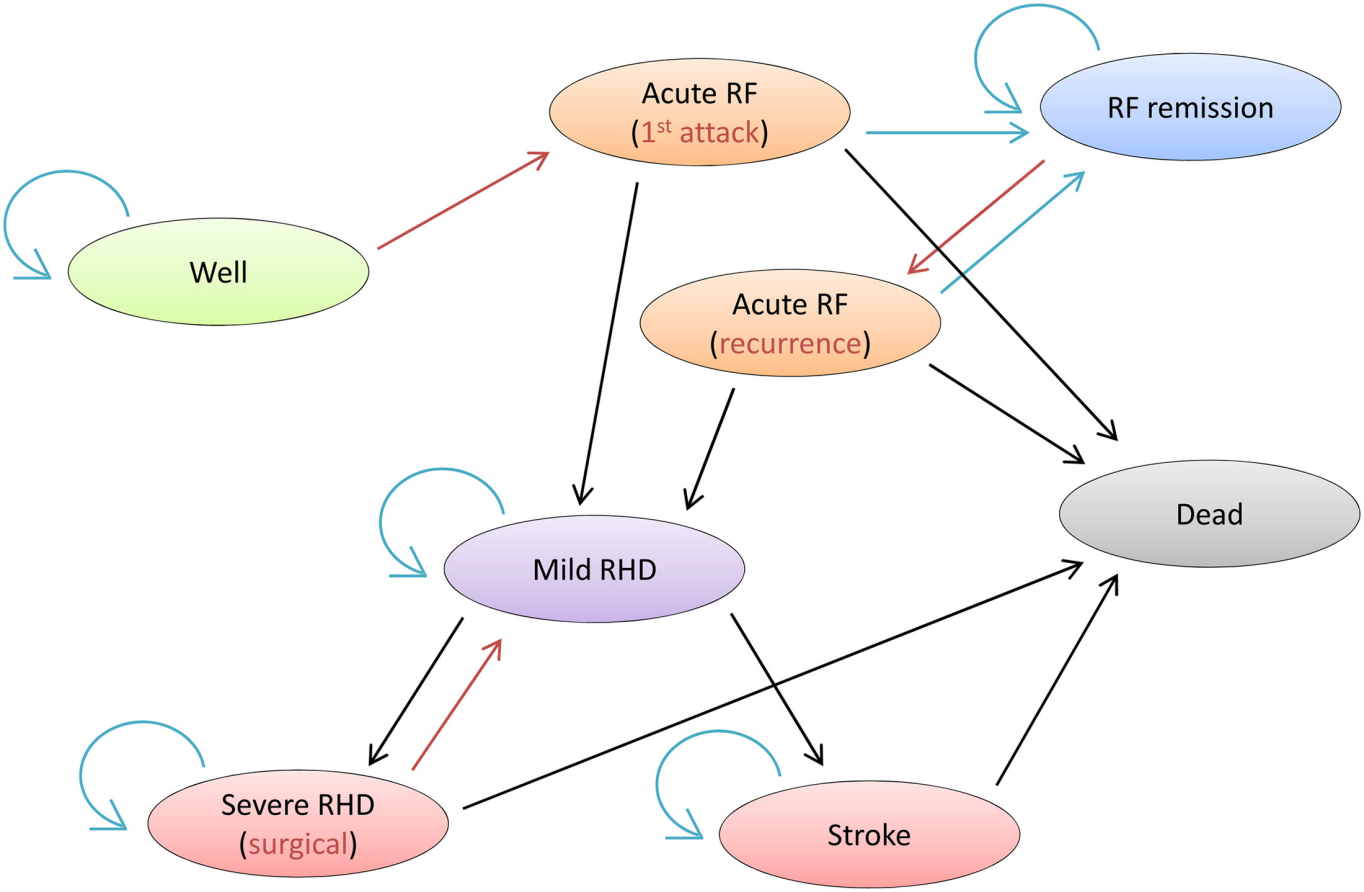
Schematic of the Markov model developed for this study.

We developed a Markov model that includes the most important health states across the natural history of ARF and RHD. The model was developed from clinical experience, available epidemiological data, and expert opinion in cases where data on transitions between states are less clear. Fig 1 depicts the model and highlights, using red arrows, the transition probabilities that are impacted by the three intervention scenarios.

The Markov traces used weighted average transition probabilities that accounted for the intervention coverage before and after the intervention was scaled up. For instance, if VS coverage is assumed to be 10% ante and 95% post, then the transition probabilities would be

$${\text{TP}}_{\text{ante}}=10\%\;\times \;\left({\text{TP}}_{\text{surgery}}=80\text{\%}\right)+95\%\;\times \;\left({\text{TP}}_{\text{no surgery}}=0\text{\%}\right)=8\text{\%}$$

and

$${\text{TP}}_{\text{post}}=95\%\;\times \;\left({\text{TP}}_{\text{surgery}}=80\text{\%}\right)+10\%\;\times \;\left({\text{TP}}_{\text{no surgery}}=0\text{\%}\right)=76\text{\%}$$

The choice of coverage rates will be an important consideration for analysts using this model. Realistic target coverage rates should be selected in consultation with public health and clinical experts on RHD. In general, we recommend lower coverage rates for PP than for SP and VS, since identification and treatment of all cases of streptococcal pharyngitis is practically more challenging than providing SP or VS.

For PP (i.e., transition from “Well” to “Acute RF”), and for SP (i.e., transition from “RF remission” to “Acute RF”), our transition probabilities were based on effect sizes from systematic reviews (PP and SP) of clinical trials.[18,19] Because there have been no controlled studies of VS, our transition probability was based on long-term survival rates from case series of mitral valve replacement.[20] Consistent with clinical experience, we assumed no remission from the severe RHD state in the absence of the surgical intervention.

#### Epidemiological inputs

The effect sizes of the interventions, the other transition probabilities used the model, and the disability weights used for the health outcomes are detailed in Table 1. Many of the transition probabilities are based on historical data or expert opinion, so where the literature did not provide estimates of uncertainty, we chose low and high bounds that were 33% higher or lower than the point estimates in the literature.

**Table 1:** Transition probabilities used in the model.

Transition	Base	Low	High	PSA distribution	Source
Chance of ARF (first episode) *	0.00045	0.00030	0.00060	beta	Irlam[13]
Chance of progression to RHD (ARF first episode)	0.360	0.241	0.479	Dirichlet	Hewitson[21]
Case-fatality rate from ARF (first episode)	0.010	0.005	0.020	Dirichlet	Irlam[13]
Chance of ARF (recurrence) **	0.113	0.075	0.150	beta	Hewitson[21]
Case-fatality rate from ARF (recurrence)	0.020	0.010	0.040	Dirichlet	Assumption
Chance of progression to RHD (ARF recurrence)	0.720	0.482	0.958	Dirichlet	Hewitson[21]
Chance of progression to chronic HF	0.008	0.005	0.011	Dirichlet	Michaud[15]
Chance of remission from chronic HF	0.000	0.000	0.000	Dirichlet	GBD 2013[5]
Chance of death from HF	0.125	0.088	0.166	Dirichlet	Gunther[7]
Chance of developing AF and stroke	0.003	0.002	0.004	Dirichlet	Sliwa[22]
Chance of death given stroke	0.167	0.130	0.190	beta	Feigin[23]
Risk reduction from primary prevention	0.320	0.210	0.480	lognormal	Robertson[18]
Risk reduction from secondary prevention	0.450	0.220	0.920	lognormal	Manyemba[19]
Risk reduction from valve surgery	0.800	0.690	0.910	lognormal	Zuhlke[20]
ARF disability weight	0.005	0.003	0.007	beta	GBD 2013[5]
RHD disability weight	0.041	0.026	0.062	beta	GBD 2013[5]
HF disability weight	0.179	0.122	0.251	beta	GBD 2013[5]
Stroke disability weight	0.070	0.046	0.099	beta	GBD 2013[5]
Average age of ARF first attack	8	5	11	gamma	Assumption
Average age of ARF recurrence	12	8	16	gamma	Assumption
Average age of RHD prevalence	24	14	34	gamma	Assumption

* cycles 5–14 only;

** cycles 5–44 only

We made three major assumptions because of a lack of published data. First, we assumed that the case-fatality rate from recurrent episodes of ARF was twice that of initial episodes. Second, we assumed that the average ages of initial ARF, recurrent ARF, and symptomatic RHD were 8, 12, and 24 respectively. Third, we assumed that the highest probability of initial and recurrent ARF occurred during ages 5–14 and 5–24, respectively, and that the probability decayed exponentially thereafter such that the transition probability *t* years later (TP_*t*_) as compared to the baseline risk (TP_0_) is

$${\text{TP}}_{\;t}=\text{TP}{\;}_{0}\;\times \;e^{-0.1\left(\;\Delta \;t\right)}$$

Advanced analysts can edit these assumptions, as well as the literature-based inputs, if local data are available. Additionally, follow-up data from the 12-country REMEDY study are forthcoming and can be used to calibrate the model to local or regional patterns.[9] Additionally, the key parameter driving PP—incidence of ARF—can (and ideally should) be input by the analyst following a needs assessment that includes a local estimate of the incidence of ARF.

#### Health outcomes

In this analysis, we used a utility-based measure of health outcomes, disability-adjusted life-years (DALYs). The DALYs averted in each of the intervention scenarios represents the improvement in health for the reference case in that scenario that would result from increased coverage of the intervention; i.e., the difference in the weighted average probability that the individual will receive the intervention. To calculate the years of life lost (YLL) component of DALYs, our model allows for input of country-specific life tables, and we subtract background mortality from the YLLs in the ante and post traces. To calculate the years lived with disability (YLD) component of DALYs, we used disability weights from the most recent Global Burden of Disease study.[5] We modeled all health outcomes over a lifetime horizon (100 one-year cycles) and discounted at the standard 3% per year.

### Program and healthcare cost inputs

The anticipated end user of this tool is the ministry of health, so our analysis takes a health system perspective on costs rather than a societal perspective. The model is designed to estimate healthcare costs over a lifetime horizon (100 one-year cycles) and discounted at the standard 3% per year. The three intervention scenario program costs are designed to be calculated on a separate worksheet and estimated over a time horizon that is appropriate to each intervention, again, discounted at 3%.

The worksheet is designed so that the end user can, prior to running the model, estimate the program costs that would be required in order to achieve full coverage of PP, SP, and VS. In general, the cost of scaling PP includes community and provider education, surveillance, program administrative costs, and additional clinical expenses needed to manage all cases of streptococcal pharyngitis appropriately. The cost of scaling SP includes case finding efforts, maintenance of a patient registry, provider education, program administrative costs, and additional clinical expenses needed to deliver monthly penicillin injections to all cases.[24]

Estimating the cost of scaling VS is somewhat more challenging and depends on the present availability of specialized surgery in the country. On the one hand, some ministries of health may wish to build local surgical capacity, so their costing exercise would focus on the capital and recurrent costs of a specialized health facility. On the other hand, other ministries of health may have good international relationships with centers that do a high volume of RHD surgeries (e.g., Sudan and India). These ministries may wish instead to scale VS through a program to find all potential surgical candidates and facilitating their surgery abroad while sharing costs with the host country. So their costing exercise would focus on case-finding efforts, patient travel, and any surgical costs borne by the local country government. We explore both of these choices in our example below.

### Application of the model

To illustrate how this model can be used to set local priorities, consider a hypothetical African country with low (10%) baseline coverage of PP, SP, and VS. This country has approximately 4.9 million population aged 5–24 years who are at highest risk of RHD (total population 20.9 million). The country’s life expectancy at birth is 68 years and its per capita gross domestic product is US$ 1300. Its government currently spends $80 per capita on health. The crude prevalence of RHD in the pediatric population is 1%, and the cumulative incidence of ARF in this group is 1.7% (back-calculated from an assumed 5.7% lifetime cumulative incidence of ARF).[25] The ministry of health in this country is considering the cost-effectiveness of scaling up PP, SP, and VS.

For PP, the ministry’s target coverage rate is 70%. This low target coverage rate reflects a major challenge of PP, which is that a significant fraction of individuals with ARF do not seek care for sore throat even in the best of circumstances.[6] The objective of the PP program is to educate the community on ARF and strengthen primary care services, but the imperfect coverage reflects the realistic effectiveness of the intervention rather than its efficacy.

For SP, the ministry’s target coverage rate is 92%. This coverage rate assumes that all individuals with a history of ARF are identified and enrolled in a registry and that these individuals are adherent to 11 of 12 of their monthly penicillin injections (i.e., they miss on average one dose each year). The SP program is relatively more expensive per patient than PP because of the human resources required to find cases and maintain a registry. We took PP and SP costs from the aforementioned publication of a combined PP and SP program in Cuba.[3] However, we estimated the program cost over 30 years instead of the 10 years originally published. We chose this longer time horizon for two reasons: first, to coincide with the useful life of a surgical center (see below), and second, because experience from high-income countries suggests prevention efforts would need to be sustained over decades to achieve true “eradication.”[1]

The ministry has two options for VS. There is currently no surgical capacity in country, and the 10% of eligible individuals who have undergone surgery in recent years have all traveled to India and have been self-financed. The first option the ministry is considering is to build a local, high-volume, specialized surgical center. The second option is to invest heavily in getting all eligible individuals to surgery in India. In either case, the target is near-universal (95%) coverage.

The costs of these two VS options are estimated differently. Building a surgical center will require about US$ 20 million in capital investments and US$ 300,000 per year in recurrent operating costs (including the direct cost of surgery as well as other activities such as case-finding and case management).[26] Assuming this center has a 30-year lifespan, the annualized capital cost would be about $970,000. The sum of the annualized capital and recurrent costs is the cost of the program in our model, calculated on a per capita basis.

For the alternative, i.e., leveraging international surgical centers, there would be two components to the program cost. First, achieving universal coverage would involve case finding activities and referral of all possible surgical candidates; we assume this to be 50% of the per capita cost of the SP program. Second, the government would need to pay the Indian government for the operations themselves. Assuming the marginal cost of valve surgery to be $5000, with 1000 surgeries performed a year (as in the first option), and a 5% administrative cost, the cost per prevalent RHD case would be about $129 per year. Table 2 outlines the healthcare and program costs used in the illustration.

**Table 2:** Healthcare and program costs used in this analysis.

Cost	Base	Low	High	Source
ARF hospitalization	$1490.00	$745.00	$2235.00	Watkins[24]
Remission (secondary prevention)	$288.00	$144.00	$432.00	Watkins[24]
RHD (secondary prevention)	$617.10	$308.55	$925.65	Watkins[24]
Severe HF	$957.10	$478.55	$1435.65	Watkins[24]
Stroke and AF	$617.10	$308.55	$925.65	Assumption
Primary prevention component	$3.66	$1.83	$5.48	Watkins[24]
Secondary prevention component	$2226.59	$1113.30	$3339.89	Watkins[24]
Scale up of surgery: build center	$25,626.54	$12,813.27	$38,439.80	Assumption
Scale up of surgery: refer abroad	$3711.69	$1855.84	$5567.53	Assumption

Costs in 2010 US dollars; all costs discounted at 3%

### Analyses undertaken

In the base case scenario, we estimated ICERs for PP, SP, and VS (both options discussed previously). We also conducted a univariate sensitivity analysis on all model inputs and present tornado diagrams of the ten inputs that were most influential on the ICERs. Finally, we conducted a probabilistic sensitivity analysis on all model inputs simultaneously over 2000 trials. Distributions used for the transition probabilities are given in Table 1. All costs (Table 2) were drawn from gamma distributions with high and low values that were 50% of the base case values. Both the univariate and probabilistic sensitivity analyses are preprogrammed into the model spreadsheets as Excel macros.

## Results

### Base case analysis

Table 3 presents in a league table the ICERs for the four potential interventions (including two alterative approaches to VS) in the hypothetical country described above. Scaling PP would be cost saving, and scaling SP would be cost-effective in the base case but with a very wide 95% credible interval. Scaling VS would not be cost-effective at usual thresholds of one to three times GDP per capita; however the international referral approach would be cost-effective at a threshold of less than three times GDP per capita.

**Table 3:** Results of base case analysis.

Scenario	ICER (mean)	ICER (95% CI)	Multiple of GDP per capita	Increase in healthy life expectancy
Scale up PP	-$2539.01	-$2952.26 to -$2003.27	---	0.25
Scale up SP	$752.34	-$540.35 to $21,671.14	0.6	19.46
VS: build surgical center	$23,827.04	$21,372.47 to $26,746.79	18.3	8.57
VS: refer for surgery abroad	$3814.41	$3154.24 to $4675.60	2.9	8.57

ICERs expressed as cost per DALY; costs in 2010 US dollars; all costs and outcomes discounted at 3%; CI = credible interval; GDP = gross domestic product

The three interventions would also result in differential gains in population health. Extrapolating to the entire population, scaling PP, SP, and VS would avert 501, 1025, and 218 total DALYs each year, respectively. These would translate into an increase in healthy life expectancy of 0.25 years for the general population, 19.5 years for individuals with a history of ARF, and 8.6 years for individuals with RHD, respectively.

### Univariate sensitivity analyses

**Fig 2:**
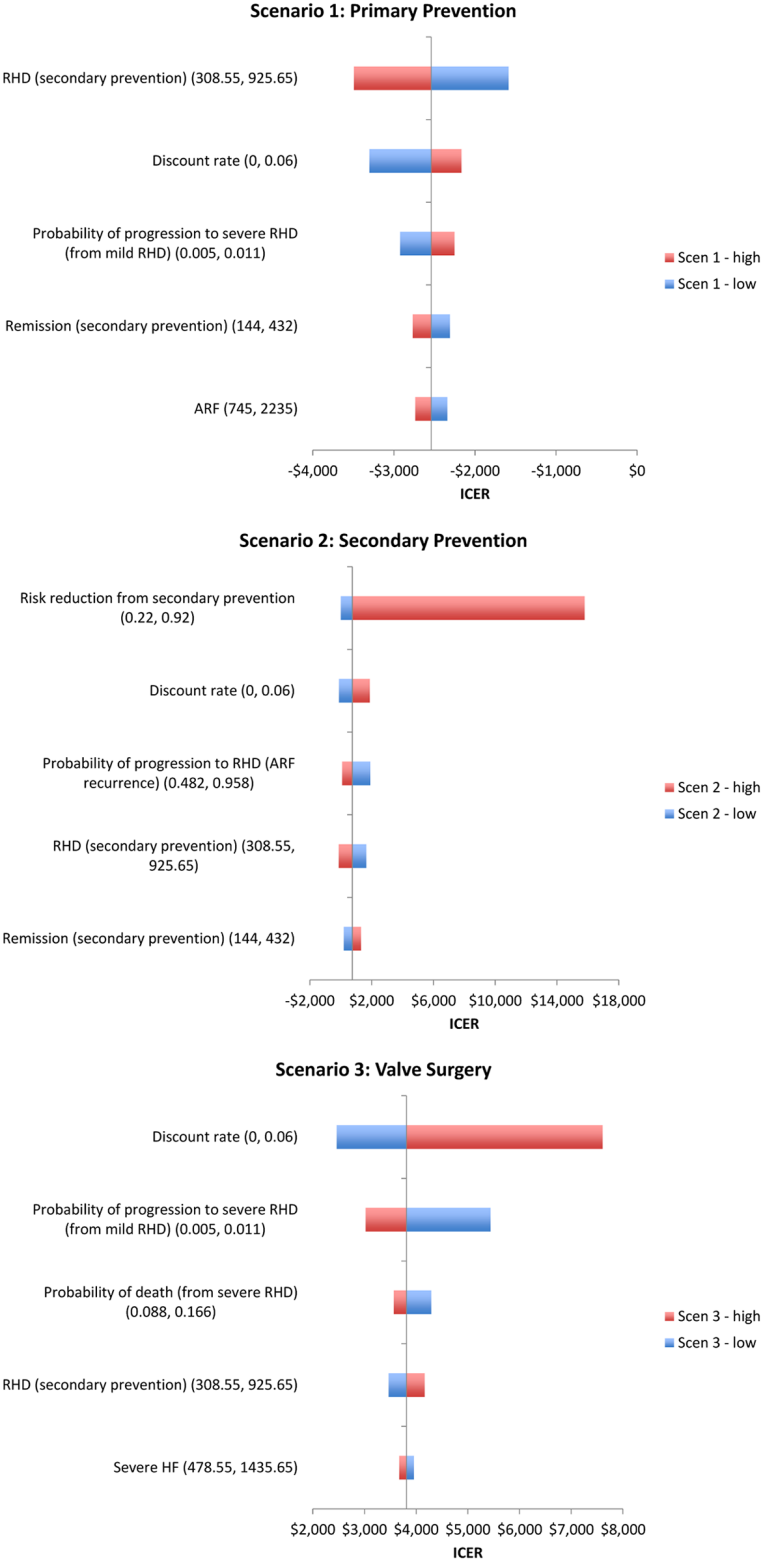
Tornado plots of the univariate sensitivity analyses for the 3 scenarios. Note that the two VS approaches were similar to the same sets of inputs, so only the second (international referrals) is shown.

Relatively speaking, each scenario in our model was sensitive to different sets of inputs (Fig 2). For PP, the cost of secondary prevention, the discount rate, and the progression rate from mild to severe RHD were the most influential inputs. For SP, by and far the most influential input was the risk reduction from secondary prevention, which is reflected in the wide 95% credible interval for the ICER (Table 3). For VS, the discount rate and the progression rate from mild to severe RHD were the most influential inputs.

### Probabilistic sensitivity analyses

**Fig 3:**
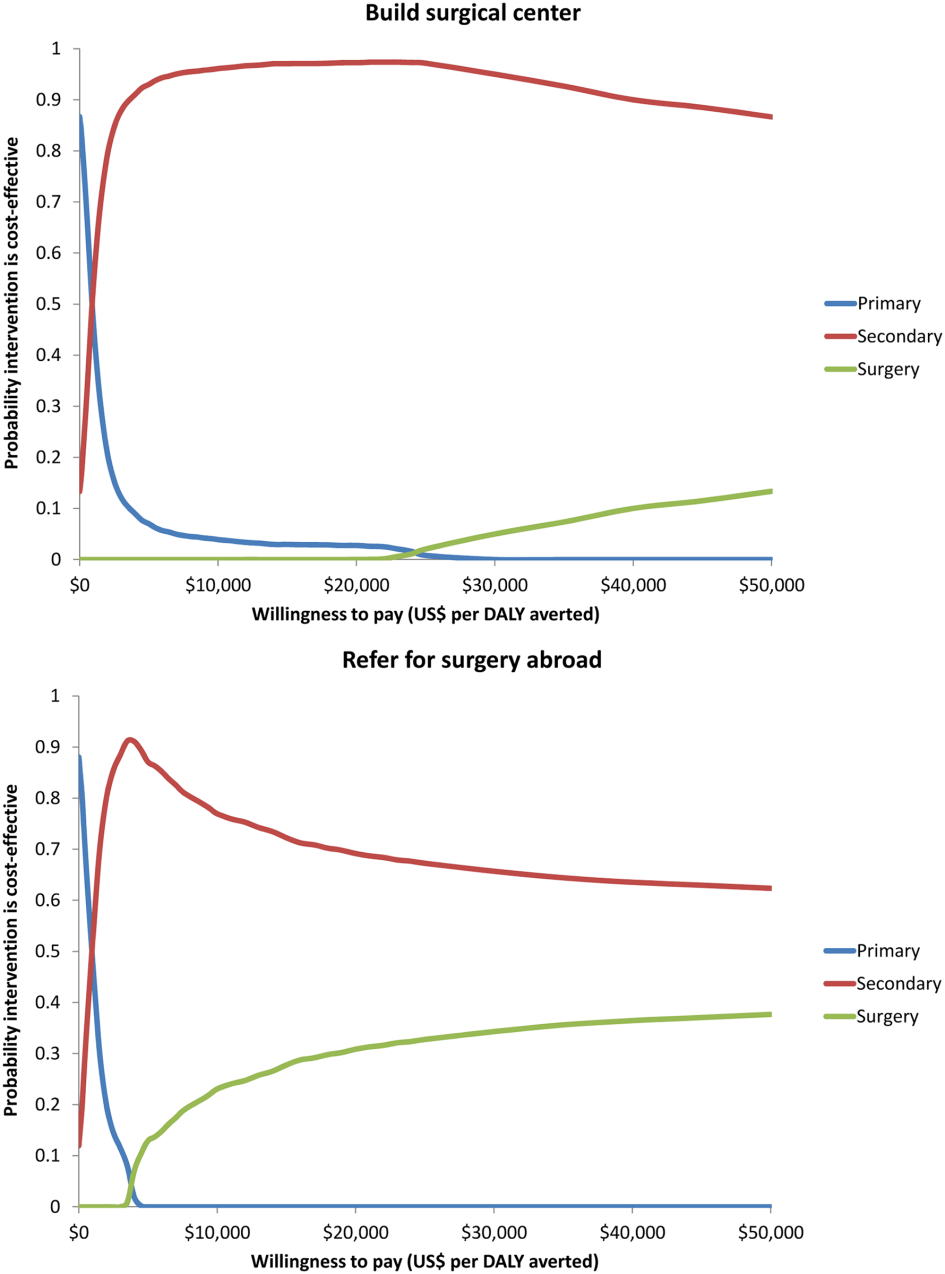
Cost-effectiveness acceptability curves.

When all input parameters were varied simultaneously, PP was the most acceptable intervention at the lowest levels of willingness to pay. SP became acceptable vs. PP above approximately $1000 per DALY. VS was not acceptable vs. SP at a maximum willingness to pay threshold of $50,000 per DALY; however, the probability of VS being acceptable was much higher if the international referral approach was taken as compared to the approach of building local surgical capacity. The large difference in program costs was the distinguishing factor in the VS cases as the health gains were the same for either approach. Fig 3 presents cost-effectiveness acceptability curves for the three scenarios.

## Discussion

We present a flexible economic evaluation tool that can be used to set priorities around RHD prevention and control in endemic, limited resource settings. This tool can allow ministries of health to allocate resources more efficiently by comparing beforehand the potential value for money of different RHD interventions. Recognizing that public health programs must now intersect with the Sustainable Development Goals, our analysis centers on the health and economic impact of achieving universal coverage for various RHD interventions.[27]

Our hypothetical case study suggests that achieving universal PP coverage in particular can greatly improve population health and result in cost savings. These potential savings are an especially important consideration for governments looking for fiscal space to expand their benefits packages over time. Our findings contrast with the longstanding belief that SP is the most cost-effective approach to RHD control. This belief emerged from a limited number of economic evaluations conducted in the 1980s and early 1990s.[15,28] Our analysis, which uses more up-to-date data, corroborates the concern expressed by some experts that PP has been inappropriately neglected by ARF and RHD control programs.[29]

At the same time, it should be noted that achieving full coverage of PP is infeasible in most settings, since many patients with RHD (as many as 50% in one report from the USA) do not recall a history of ARF.[30] Our model has been designed to account for inefficiencies in PP. For example, if local experts believe that only 50% of ARF cases can realistically be prevented, then the analyst could use a target coverage rate of 50% rather than 70% (our assumption) or higher. When this coverage rate is changed in our model and all other parameters are held constant, the ICER is slightly higher because of lower health gains for the same cost; however, the PP intervention is still cost saving.

While our hypothetical country application is illustrative rather than prescriptive, a few general conclusions emerge from this exercise. First, programs to prevent RHD are probably more cost-effective than programs to treat RHD (i.e., by surgery). This should come as no surprise to clinicians and public health practitioners who deal with ARF and RHD; advocates have long pointed out that, amongst non-communicable diseases, RHD is uniquely preventable and even eradicable over time—by contrast, e.g., to ischemic heart disease.[1] However once established, RHD carries high rates of morbidity and mortality among children and working-age individuals.[6,7]

Second, our model identifies important data gaps that should be addressed in future RHD research. While the natural history of ARF and RHD is qualitatively well understood, few contemporary studies have estimated incidence and progression rates between disease states in a comprehensive manner. Hence the ICERs generated in our illustrative case are very sensitive to these inputs. High-quality data from longitudinal studies would greatly improve the precision of our model. Along these lines, RHD epidemiologists should engage health economists in their work to gather better data on the economic aspects of the disease.

Lack of data is particularly challenging in the case of SP. We have not explicitly considered echocardiography-based screening (“active case-finding”) as an approach to SP. A recent cost-effectiveness analysis determined that active case-finding was cost-effective compared to passive case-finding. However, this model was predicated on the assumption that SP for cases identified through screening is as effective as SP for clinical cases of ARF and RHD.[31] In our view, there is insufficient evidence at present to suggest that active case-finding improves outcomes, and it is certainly not known how effective active case-finding is compared to passive case-finding. Experimental or quasi-experimental studies would be required to resolve this issue. Having said this, an active case-finding scenario could readily be incorporated into our model: a decision tree would be constructed that incorporated echocardiography test performance characteristics, and this tree would lead into separate Markov traces.

Third, decisions on how to proceed with VS in very poor countries should be made carefully. Our results suggest that, for a hypothetical low-income country, building local VS capabilities may not be a good initial investment due to its high cost and limited impact on population health. If the government budget allows, however, a referral-based approach to VS may be cost-effective. By contrast, a lower- or upper-middle income country with a higher willingness-to-pay threshold (e.g., US$10,000 per DALY) might reasonably consider building a local surgical program. Regardless, our analysis suggests that resources in countries similar to our hypothetical case should be invested in PP and SP until full coverage is achieved before moving onto VS.

Aside from cost-effectiveness per se, an additional consideration for any public health intervention is affordability. While in this example PP would result in cost savings in the long run (i.e., a “negative” total incremental cost), these savings would only be realized after an up-front investment of about $874,000 per year that would rapidly reduce ARF and result in cost savings from cases of ARF and RHD averted. SP and VS would not be cost saving, however, and their annual incremental costs would be much higher–$771,000 for SP, and $831,000 to $5.2 million for VS (depending on approach). Holding government health expenditure (GHE) constant over time, scaling SP would add 0.2% to GHE for the hypothetical country. The two VS approaches (build surgical center or refer for surgery abroad) would add 1.3% or 0.2% to GHE, respectively.

While SP and VS would be expensive, the percentages listed above suggest they would not necessarily be financially unsustainable in a low-income country—particularly the less-expensive, referral-based VS approach. On the other hand, low-income countries have a large number of competing health priorities, and it may be that in any given country there are a number of interventions for conditions other than ARF and RHD that are more effective and less costly. These should, in principle, receive higher priority in the short run.[32] Still, for this particular hypothetical country, our analysis suggests that PP would be very effective and relatively inexpensive and could easily be included in any list of first-priority interventions.

It should also be noted that, although cost-effectiveness analysis is an appropriate method for evaluating PP, SP, and some sorts of VS interventions, it might not adequately address the issue of whether to build local surgical capacity. Surgical centers have important implications outside the narrow field of RHD.[33] For instance, economies of scope would likely emerge from the ability to treat, e.g., congenital heart disease or coronary artery disease at the same facility, using much the same capital and labor inputs. Such a center could also be an important hub for clinical training and scientific research, which have important non-health benefits to society. These broader economic considerations could be better accounted for in a benefit-cost analysis.[34]

There are three important limitations to our analysis. First, we did not incorporate other significant sequelae of RHD, such as maternal mortality, infective endocarditis, and specific complications of surgery.[6] The complex interactions between the various RHD sequelae would best be handled using a microsimulation approach; however, good epidemiological data for RHD are at present scarce and not of sufficient quality or detail to inform such a model. Second, although we have framed our analysis in terms of UHC, we have not attempted to incorporate some of the non-health goals of UHC, such as financial risk protection, that would be better handled in a benefit-cost analysis or extended cost-effectiveness analysis.[17] Future studies could explore these complementary analytical approaches. Finally, our analysis is greatly limited by cost data. Our hypothetical country illustration relied heavily on “best guesses” or extrapolation of costs from other parts of the world. End users of our tool will need to collect their own primary cost data to get the most out of the analysis, since there are very few studies of ARF/RHD costs in Africa from which to draw.

RHD continues to exact a high health and economic toll on African countries, but evidence-based prevention and treatment measures are currently underused. We have made available in the public domain a cost-effectiveness analysis tool that can be used at the local level to guide the scale-up of these interventions (S1 File). In the future, we will seek to gather more empirical data on the natural history of RHD and the cost of care in African countries. These data will strengthen the precision of our model and its application in limited resource settings.

## Supporting Information

S1 File RHD cost-effectiveness analysis tool. (XLSM)
